# Self-stigma and cognitive insight in individuals at ultra-high risk for psychosis

**DOI:** 10.3389/fpsyt.2023.1154284

**Published:** 2023-04-13

**Authors:** Bouwina Esther Sportel, Mirjam van Enthoven, Rozanne J. M. van Donkersgoed, Daan Jan Kuis, Tara van de Giessen, Paul H. Lysaker, Ilanit Hasson-Ohayon, Steven de Jong, Nynke Boonstra, Gerdina H. M. Pijnenborg

**Affiliations:** ^1^Department of Psychotic Disorders, GGZ Drenthe Mental Health Institute, Assen, Netherlands; ^2^Department of Clinical and Developmental Neuropsychology, Faculty of Behavioral and Social Sciences, University of Groningen, Groningen, Netherlands; ^3^Department of Psychiatry, Richard L. Roudebush VA Medical Center, Indianapolis, IN, United States; ^4^Department of Psychiatry, Indiana University School of Medicine, Indianapolis, IN, United States; ^5^Department of Psychology, Bar-Ilan University, Ramat-Gan, Israel; ^6^Lentis Psychiatric Institute, Groningen, Netherlands; ^7^KieN Early Intervention Service, Leeuwarden, Netherlands; ^8^Research Group Care and Innovation in Psychiatry, NHL Stenden University for Applied Sciences, Leeuwarden, Netherlands; ^9^Department of Psychiatry, UMC Utrecht Brain Center, University Medical Center Utrecht, Utrecht, Netherlands

**Keywords:** stigma, insight, ultra-high risk, psychosis, schizophrenia, cognitive insight, self-stigma

## Abstract

**Background:**

Impaired cognitive insight and increased self-stigma have been consistently reported in individuals diagnosed with schizophrenia spectrum disorders, but little is known about its presence in individuals at ultra-high risk of developing a psychosis, although self-stigma is associated with transition.to psychosis. The current study examined whether self-stigma is already present in individuals at ultra-high risk of psychosis, and whether this is associated with impaired cognitive insight.

**Methods:**

184 participants were recruited divided over three groups, namely individuals diagnosed with a schizophrenia spectrum disorder (SSD; n = 92, 34% females), individuals at ultra-high risk for psychosis (UHR; n = 43, 59% females) and general population controls (GPC; n = 49, 27% females). All participants completed assessments on demographic information (gender, age, education), and cognitive insight. In addition, participants with SSD and individuals at UHR completed a questionnaire on self-stigma.

**Results:**

The level of self-stigma did not differ between individuals at UHR and individuals diagnosed with SSD. Cognitive insight also did not differ significantly between the three groups, but the subscale self-reflection differed between the three groups [*F*(2,184) = 4.20, *p* = 0.02], with the UHR and SSD groups showing more self-reflection. Pearson’s correlation analyses showed that in individuals at UHR total cognitive insight and its self-reflection subscale were significantly associated with the alienation subscale of self-stigma, and in individuals with SSD self-certainty subscale of cognitive insight was significantly associated with stereotype endorsement.

**Conclusion:**

Findings show that self-stigma was already present in the UHR phase, to a similar degree as in individuals with a diagnosis of a SSD, and is thus not dependent of previous experience of having a label of SSD. Cognitive insight in individuals at UHR of psychosis appears to be intact, but individuals at UHR showed more self-reflectiveness, and individuals at risk with high cognitive insight also experience high levels of self-stigma. Overall findings from our study suggest that pre-emptive interventions targeting self-stigma, while considering cognitive insight, are needed early on in manifestation of psychotic illness, preferably already in the UHR phase.

## 1. Introduction

Stigma associated with mental disorders is highly prevalent worldwide and is considered a public health problem given the consequences of stigma (1). Schizophrenia spectrum disorders (SSD) are among the most stigmatized in society (prevalence rate of 18–71%), compared to depression (14–33%) or anxiety disorders (26%) (2, 3).

Stigma includes elements of labeling, stereotyping, separation, status loss, and discrimination which often unfold within the context of a perceived power differential (4). Stigma of mental illness can be distinguished into public stigma, where the general population endorses prejudice and manifests discrimination toward people with mental illness, and self-sigma (5), where public stigma is internalized, meaning that the person applies the negative stereotypes about mental illness to himself or herself. Self-stigma is relatively common in people with an SSD with 36.9% of patients reporting high levels of self-stigma according to a systematic review (6). Self-stigma is associated with multiple negative outcomes, such as lower levels of cognitive insight (7, 8), low self-esteem, less hope (9, 10), higher suicide risk (10), reduced self-efficacy, less empowerment (6, 11–14), and worse subjective well-being (15). It is also shown to be longitudinally related to a variety of negative outcomes including more positive symptoms, anxiety and depression, lower self-esteem, and poorer social functioning (6), and self-stigma can play a mediating role between negative symptoms and recovery (16).

Interestingly, self-stigma was shown to be related to several self-reflective processes, such as Theory of Mind, clinical insight and metacognition (17–19). Moreover, a negative association between self-clarity, i.e., having a clear and positive sense of self, with self-stigma was shown (20). The few studies that focus on general reflective processes suggest that being more reflective may facilitate the development of a richer personal narrative and as such buffers against self-stigmatizing generic beliefs about one’s identity. Other studies that focus on clinical insight, i.e., being aware of having an illness, symptoms of this illness and need for treatment (as opposed to more general awareness), show a positive association between clinical insight and self-stigma (19). Thus, different conceptualizations and methods of assessment of reflection show different associations with self-stigma. All of these studies were conducted among patients who already received a diagnosis of a severe mental illness. Little is known about self-stigma levels and about the relationship between self-stigma and reflective processes in individuals with an ultra-high risk for psychosis, who are by definition not yet diagnosed with an SSD.

Psychosis occurs along a continuum ranging from psychotic experiences in the general population and subclinical symptoms in the UHR phase, to more distressing crystallized symptoms in SSD (21). It is possible that self-stigma is present in the earlier phases as well, starting from the moment where first symptoms occur and label and treatment is introduced, and one’s own awareness is raised. Gaining more insight in the presence of self-stigma in the UHR phase is important, as it has been identified as a risk factor for the transition to a first episode of psychosis (22). A recent systematic review shows that individuals at UHR of psychosis experience more self-stigma than healthy subjects (23). However, the seven studies included in this systematic review widely differ in their design, used instruments and included groups of participants. In addition to a paucity of studies on the level of self-stigma in the UHR population, to the best of our knowledge, no studies assessed the association of self-stigma with reflective processes in this population.

As mentioned above, there are different conceptualizations of reflective processes. One interesting concept presenting reflection is cognitive insight, which is not necessarily indicative of whether individuals recognize their mental illness (as with clinical insight), but refers to a reflective process of reevaluating their experiences, and correcting of distorted beliefs and misinterpretations (24). Cognitive insight is therefore the ability to distance oneself from psychotic experiences, to reflect on them and to make use of feedback from others to re-evaluate one’s own interpretations (25). Research implies that cognitive insight is a better indicator of prognosis following CBT (25), and is also less prone to inflation by learning to provide the right responses over time (24). In addition, given that individuals who are at ultra-high risk of psychosis do not have a diagnosis of a schizophrenia spectrum disorder (yet), clinical insight might not be an appropriate nor relevant assessment during this phase, and cognitive insight is a more suitable measure instead. As such, the current study focusses on cognitive insight, rather than clinical insight.

Research has shown that cognitive insight is significantly lower in people with an SSD compared to people with a mental disorder without psychotic symptoms (24, 25). However, within the UHR population, cognitive insight has received considerably less attention. It is therefore still unclear whether cognitive insight is impaired, or intact, in the UHR population, as compared to individuals in the general population and people with schizophrenia. A meta-analysis including five studies has been conducted on this topic, finding higher idiosyncratic self-certainty, a subscale of cognitive insight, in an at-risk population compared to a general population sample, but no differences on the self-reflectiveness subscale or overall cognitive insight (26). A few studies not included in the meta-analysis focused on the differences between an UHR population, people with SSD, and general population controls, finding some conflicting results (27–29). Uchida et al. (28) found, partly in line with Dondé et al. (26), lower cognitive insight (specifically higher self-certainty) in the UHR population to a similar degree as in SSD, and significantly lower than general population controls. This in contrast to two other studies (27, 29), that found intact cognitive insight in the UHR population as compared to general population controls, and significantly higher cognitive insight as compared to individuals with a diagnosis of schizophrenia.

To date, no study has been conducted investigating the relationship between cognitive insight and self-stigma among individuals with UHR. Thus, the aim of the present study is to examine (a) the level of self-stigma in the UHR phase, as compared to individuals diagnosed with an SSD, (b) the level of cognitive insight in the UHR phase, as compared to general population controls and individuals diagnosed with an SSD, and (c) the relationship between cognitive insight and self-stigma in a sample of individuals at UHR of psychosis. We expect self-stigma to be present in individuals at UHR of psychosis, but significantly lower than in individuals with an SSD. We expect that cognitive insight will be higher in individuals at UHR of psychosis than in individuals with an SSD, but lower than general population controls. Last, it is expected to find a negative relationship between cognitive insight and self-stigma in UHR samples.

## 2. Methods

### 2.1. Participants and setting

For the current study, three groups of participants were included, namely those at ultra-high risk (UHR) for psychosis (*n* = 43), individuals diagnosed with an SSD (SSD; *n* = 92), and general population controls (*n* = 49). Data from the UHR sample and some matched general population controls (*n* = 8) were collected for the purpose of the current study, whereas data from subjects with a diagnosis of SSD and most of the general population controls (*n* = 41) originated from a randomized controlled trial (RCT) assessing metacognitive therapy in individuals with SSD (30). In this study, the general population group (aged range 18–65) was recruited through messages on social media channels (Facebook, LinkedIn), local secondary schools and school for vocational education and flyers in the villages or cities were the mental health care centers were located. All interested people were included if they met the following inclusion criteria: aged 18–65, reporting that they had never received a psychiatric diagnosis nor received treatment for mental health problems.

Inclusion criteria for the UHR sample included meeting the UHR criteria of the Comprehensive Assessment of At Risk Mental State [CAARMS (31, 32)]. CAARMS criteria consist of: the presence of mild but persistent psychotic symptoms in the past year, the experience of short-lived (<1 week) psychotic symptoms (brief limited intermittent psychotic symptoms) or a first degree relative diagnosed with SSD. In addition, participants had to be aged between 15 and 35 years old and social functioning had to be impaired [a drop in functioning in the last year of at least 30% as measured with the Social and Occupational Functioning Scale (33)]. Exclusion criteria were the presence of a co-morbid neurological disorder, substance dependence or an estimated intellectual disability (IQ < 70). Participants were recruited from two mental health care organizations in the Netherlands: Drenthe Mental Health Care Services and Friesland Mental Health Care Services. Almost all eligible clients were invited to participate by their practitioner a few weeks after UHR status was established, due to parallel studies not all were invited. The study was granted permission from the medical ethics committee of the University Medical Center Groningen (UMCG; ref. no. NL49321.042.14).

Participants diagnosed with an SSD all took part in the “Metacognition Reflection and Insight Therapy (MERIT)” study (34) which has also been authorized by the medical ethics committee of the University Medical Center Groningen (UMCG; ref. no.METc2013.124). Inclusion criteria were impaired metacognitive skills, as measured by the Metacognition Assessment Scale-Abbreviated (35), diagnosis of schizophrenia or schizoaffective disorder according to DSM-IV-TR criteria, being able to give informed consent, 18 years or older, and no change in medication in the past 30 days. Exclusion criteria were acute psychosis (mean score of PANSS positive symptoms >4), co-morbid neurological disorder, substance dependence, and impaired intellectual functioning (IQ <70). The patients in this group were recruited across seven mental healthcare institutes in the Netherlands. Eligible clients were informed about the study by their practitioner, they were given time to think about participating and were included if they were willing to participate. In total 376 clients were screened, of them 90 indicated they could not find the time to participate, 153 were not motivated to take part in the study, 30 were not stable enough, and 20 clients gave other reasons. Of the participants in the SSD group only baseline data was used.

All participants included in this study signed a written informed consent form prior to the investigation. In cases of participants under 18 years of age, legal guardians also had to give written permission.

### 2.2. Assessment of self-stigma

Self-stigma was assessed using the Internalized Stigma of Mental Illness scale [ISMI (36)], a 29-item self-report scale that includes statements related to self-stigma which participants rate using a Likert scale ranging from 1 (strongly disagree) to 4 (strongly agree). The ISMI contains five subscales, namely: Alienation, Discrimination Experience, Social Withdrawal, Stereotype Endorsement and Stigma Resistance. The test–retest reliability, internal consistency, convergent and divergent validity of the ISMI are reported as good to very good (36). However, the subscale Stigma Resistance scores is reported to have poor internal consistency. In line with previous recommendations (14, 37), Stigma Resistance was not included in analyzes. Given the nature of the questionnaire, self-stigma was not measured in the general population sample.

### 2.3. Assessment of cognitive insight

Cognitive insight was assessed using the Beck Cognitive Insight Scale [BCIS (24)], which is measured using two domains: self-reflectiveness and self-certainty. Cognitive insight is the ability to reflect on experiences and to make use of feedback from others to re-evaluate one’s own interpretations The cognitive insight index score is determined by subtracting the “self-certainty” scale from the “self-reflectiveness” scale (24). The BCIS consists of 15 self-report items, measured on a 4-point scale from 0 (do not agree at all) to 3 (agree completely). The subscale “self-reflectiveness” includes 9 items representing the ability to reevaluate experiences and correct distorted beliefs and misinterpretations, and the subscale “self-certainty” includes 6 items, measuring one’s tendency to be overconfident about one’s judgment. The validity and reliability of the questionnaire has been reported to be good (38). The cognitive insight index as well as the two sub-domains will be examined.

### 2.4. Statistical analysis

Statistical analyzes were conducted using SPSS statistical software, version 28. In order to examine whether demographic information differed between the subsamples, chi-squared analyzes were run for categorical variables (gender and education) and one-way ANOVA for continuous variables (age). Differences were found for gender and age (see 3.1 Descriptive statistics), both these variables were therefore added as covariates in the analyzes, Differences in self-stigma, total and subscales, between individuals at UHR for psychosis and individuals with SSD were assessed using a one-way ANCOVA, whilst controlling for age and gender. Differences in level of cognitive insight, and subdomains self-reflectiveness and self-certainty, between all three groups (GPC, UHR and SSD) was assessed using a one-way ANCOVA, also controlling for age and gender. Finally, a Pearson’s correlation analysis was run to investigate the relation between cognitive insight and self-stigma in individual at UHR and individuals with SSD. A Bonferroni-Holm correction was applied to correct for multiple testing. Before running the analyzes, all assumptions were checked. A violation of assumptions was found for the following subscales; ISMI Discrimination Experience, Social Withdrawal, Stereotype Endorsement, and BCIS self-reflectiveness and self-certainty. Instead of ANCOVA we used the Quade non-parametrical ANCOVA for these variables, instead of Pearson’s we used Spearman’s rho.

## 3. Results

### 3.1. Descriptive statistics

In the current study (*N* = 184), 49 general population controls were compared to 43 participants with UHR status and 92 participants with a diagnosis of SSD. Demographic information per group is shown in Table 1. In the SSD group self-estimated age of first psychosis ranged from 12 to 52 years old (*M* = 24.3, SD = 8.1), and illness duration ranged from 3 to 31 years (*M* = 16.7, SD = 9.5). Of the participants 76.1% was unemployed. Different kinds of medication were used in the past 2 weeks, including olanzapine (*n* = 14), clozapine (*n* = 12), aripiprazole (*n* = 9), lorazepam (*n* = 8), and lithium (*n* = 8). There was a significant difference in the percentage of females per group. The UHR group had significantly more females than the group of general population controls (OR: 3.16) and the group of individuals diagnosed with SSD (OR: 2.49). The general population controls and the SSD group did not significantly differ from each other with regard to gender. There was a significant difference in age between the groups, with individuals in the UHR group being significantly younger in comparison to general population controls (mean difference: 14.05, *p* < 0.01) and participants with SSD (mean difference: 16.83, *p* < 0.01). Level of education was not significantly differently distributed among the three groups. All analyzes have therefore only been adjusted for gender and age.

**Table 1:** Demographic information of study participants per subgroup (*N* = 184).

	GPC (n = 49)	UHR (n = 43)	SSD (*n* = 92)	Differences between groups
Gender, *n* (%female)	14 (28.6)	24 (55.8)	31 (33.7)	*X*^2^(2) = 8.39, *p* < 0.02
Age, M (SD)	36.14 (14.04)	22.09 (5.77)	38.92 (11.01)	*F*(2,181) = 35.30, *p* < 0.01
Education, *n* (%)				*X*^2^(4) = 5.67, *p* = 0.23
Low	14 (28.6)	14 (32.6)	33 (35.9)	
Middle	19 (38.8)	21 (48.8)	28 (30.4)	
High	16 (32.6)	8 (18.6)	31 (33.7)	

GPC, General Population Controls; UHR, Ultra-High Risk; SSD, Schizophrenia Spectrum Disorder.

### 3.2. Self-stigma in participants with UHR and SSD

Self-stigma total score and subscales scores for each group are reported in Table 2. After adjusting for gender and age, no significant difference was found between the SSD group and the UHR group on total self-stigma nor on any subscales of self-stigma.

**Table 2:** Self-stigma and cognitive insight in general population controls, participants with UHR status and participants with a schizophrenia spectrum disorder (*N* = 184).

	GPC (*n* = 49)	UHR (*n* = 43)	SSD (*n* = 92)	Differences between groups (ANCOVA/Quade)
Total self-stigma	-	2.11 (0.48)	2.18 (0.47)	*F*(1,135) = 0.54, *p* = 0.46
Alienation	-	2.38 (0.61)	2.44 (0.62)	F(1,135) = 0.25, *p* = 0.62
Stereotype endorsement	-	1.68 (0.45)	1.88 (0.46)	*F*(1,133) = 0.10, *p* = 0.76
Experience of discrimination	-	2.00 (0.68)	2.18 (0.56)	F(1,133) = 0.23, *p* = 0.63
Social withdrawal	-	2.42 (0.60)	2.26 (0.54)	F(1,133) = 1.40, *p* = 0.24
Cognitive insight	3.49 (5.10)	5.30 (5.87)	5.45 (5.34)	F(2,184) = 2.32, p = 0.10
Self-reflectiveness	11.84 (3.61)	13.88 (4.09)	13.86 (4.33)	F(2,181) = 3.786, *p* = 0.03
Self-certainty	8.35 (2.75)	8.58 (3.57)	8.41 (2.89)	F(2,181) = 0.11, *p* = 0.90

Adjusted for gender and age. GPC, General Population Controls; UHR, Ultra-High Risk; SSD, Schizophrenia Spectrum Disorder.

### 3.3. Cognitive insight in participants with UHR, participants with SSD and general population controls

Averages of cognitive insight, self-reflectiveness and self-certainty are reported per subgroup in Table 2. After adjusting for gender and age, a significant difference between groups was found for self-reflectiveness using Quade non-parametrical ANCOVA, but not for cognitive insight or self-certainty. Further investigation showed a difference between SSD and GPC [*t*(181) = −2.75, *p* < 0.01], but no differences between UHR and GPC [*t*(181) = −1.66, *p* = 0.10] or UHR and SSD [*t*(181) = 0.76, *p* = 0.45].

### 3.4. The association between cognitive insight and self-stigma in participants with UHR and SSD

A series of correlations were calculated to explore the association between cognitive insight and subscales and self-stigma and subscales in participants in the UHR group and SSD group. For participants in the UHR group moderate positive significant correlations were found for BCIS self-reflectiveness with the ISMI Alienation subscale [*r* = 0.36, *p* = 0.02, CI (0.07,0.60)], and cognitive insight with the ISMI Alienation subscale [*r* = 0.35, *p* = 0.02, CI (0.05,0.59)], and BCIS self-certainty with the ISMI Social withdrawal subscale [Spearman’s *rho* = 0.30, *p* = 0.05, CI (−0.06,0.51)]. In the SDD group we only found a small significant positive correlation between BCIS self-certainty and ISMI Stereotype Endorsement [Spearman’s *rho* = 0.22, *p* = 0.04, CI (0.00,0.40)]. No other significant correlations were found in either group (Table 3).

**Table 3:** Correlations between cognitive insight and stigma in participants with UHR status (*n* = 43) and participants with a schizophrenia spectrum disorder (*n* = 92).

Cognitive insight	Stigma	UHR	SSD
BCIS cognitive insight	ISMI total	0.04	−0.07
	ISMI Alienation	0.35*	0.03
	ISMI Stereotype endorsement^1^	0.10	−0.19
	ISMI Discrimination experience^1^	−0.10	−0.09
	ISMI Social withdrawal^1^	−0.03	0.02
BCIS self-reflectiveness^1^	ISMI total	0.17	0.00
	ISMI Alienation	0.36*	0.12	ISMI Stereotype endorsement^1^	0.04	−0.09
	ISMI Discrimination experience^1^	0.00	−0.03
	ISMI Social withdrawal^1^	0.21	0.05
	BCIS self-certainty^1^	ISMI total	0.15	0.13
ISMI Alienation		−0.02	0.11
ISMI Stereotype endorsement^1^		−0.11	0.22
ISMI Discrimination experience^1^		0.19	0.18
ISMI Social withdrawal^1^		0.30*	0.04

UHR, Ultra-High Risk; SSD, Schizophrenia Spectrum Disorder.

^1^Spearman’s rho, *p ≤ 0.05.

## 4. Discussion

The current study focused on levels of self-stigma and cognitive insight and their association in an UHR group. Findings showed that levels of self-stigma in the UHR phase are comparable to that in people who are already diagnosed with an SSD. Levels of self-reflectiveness, a subscale of cognitive insight, were significantly higher in SSD compared to general population controls, but not to UHR. It was hypothesized that more cognitive insight [i.e., the ability to reflect on experiences and to make use of feedback from others to re-evaluate one’s own interpretations (25)] would be associated with less liability to self-stigma. However, we found that the Alienation subscale of the ISMI was positively associated with self-reflectiveness subscale of cognitive insight and cognitive insight in the UHR group, Social Withdrawal was positively associated with self-certainty. In the SSD group Stereotype Endorsement, an ISMI-subscale, was positively related to self-certainty subscale of cognitive insight.

Self-stigma, as assessed with the ISMI (36), was at a similar level in individuals who are at risk for psychosis and those who have been diagnosed with an SSD. Of note, the level of self-stigma in the SSD group was in line with previous studies in comparable samples (39). It was expected that self-stigma would already be present in the UHR phase, but lower than in individuals who have already transitioned to an SSD (40). Findings of the current study show that self-stigma is not only present before the first psychotic episode, but also to a similar degree as in individuals diagnosed with an SSD. This supports previous research, which has indicated that self-stigma in the UHR phase is evident and predictive of the first psychotic episode (22), and is also higher than in patients with non-SSDs (41). Evidently, self-stigma is not dependent on having a label of a psychotic disorder. It might be that anticipating the possibility of severe mental illness in the future already leads to higher levels of self-stigma.

Cognitive insight did not differ among general population controls, individuals at risk of psychosis or individuals diagnosed with an SSD. These findings are comparable to two previous trials (28, 29), which also found no difference in overall cognitive insight between individuals at risk of psychosis and general population controls. In contrast, other studies have found that individuals with an SSD performed worse on cognitive insight in comparison to both general population controls and individuals at UHR of psychosis (27). This was not the case in our study, implying that in our clinical sample of individuals diagnosed with an SSD, cognitive insight may have been relatively intact. This is supported by the lack of difference we found in self-reflectiveness between UHR and SSD, the UHR group does not differ from SSD nor from GPC. Both UHR and SSD showed relatively high scores on self-reflectiveness compared to general population controls. A potential reason for this is that individuals with SSD and individuals at UHR in the current study have been receiving treatment at a mental health institution for a period of time, indicating their self-reflectiveness may have been improved in treatment, resulting in more cognitive insight. Previous research indicates that cognitive insight may improve after receiving CBT in patients diagnosed with schizophrenia (42) and that reductions in both delusions and hallucinations are driven by gains in cognitive insight following CBT (43).

For the UHR group, we investigated the cross-sectional relationship between cognitive insight and self-stigma to gain more knowledge into possible differences between people at UHR for psychosis in self-stigma. We found that self-reflectiveness and total cognitive insight were both significantly correlated with the alienation subscale of the ISMI scale, indicating that individuals at UHR for psychosis with higher levels of cognitive insight and self-reflectiveness, or in other words people who are more reflective while feeling a smaller certainty about being right, experience more alienation from themselves and their surroundings. We also found a positive correlation between self-certainty and social withdrawal, indicating that those who feel more certain about being right and are more resistant to being corrected, also tend to show more social withdrawal.

The association in individuals at UHR between cognitive insight and self-reflection with alienation leads to the suggestion that the impact of the at-risk status, possibly resulting in self-stigma, might be highest for those with more cognitive insight and self-reflection, future research should give more clarity on the effects of the UHR status. In our case, in individuals with UHR, cognitive insight as measured with the BCIS might not be a reflection of general awareness, but could be closer to a reflection of clinical insight, associated with more self-stigma (19). Perhaps the fact that the BCIS was filled out at the mental health facility and the questions mention ‘strange experiences’ colored the questions to be interpreted too much in relation to mental health issues and UHR status. The association between self-certainty and social withdrawal may could imply that individuals less prone to being corrected at some level choose not to have their beliefs about themselves challenged. In the SSD group we found a positive association between self-certainty and stereotype endorsement, indicating that people who feel greater certainty about being right and more resistance to being corrected also show more agreement with stereotypes surrounding mental health problems (e.g., mentally ill people tend to be violent). This is in line with recent research (44) finding an association between self-stigma and self-certainty, but not between self-stigma and self-reflectiveness. It should be noted that the confidence intervals for these correlations were quite large, and results should be replicated in a larger, independent sample. Future research looking in to the direction of this association is of importance, and might give leads to treatment focuses.

This study is the first to report on the association between reflective processes and self-stigma in a UHR sample. The current study has a number of limitations. First, although our UHR group is relatively large, the sample sizes of the different groups were still too small. A post-hoc power analysis showed that our one-way ANOVA had a power of 76% in order to detect a significant difference between the groups with a medium effect-size at a significance level of 0.05. Given that at least 80% power is recommended (45), our analyzes may have been underpowered and future studies should aim to include a bigger sample size (at least 50 participants per group). Second, the ratio female to male was different for the three groups, with an overrepresentation of women in the UHR group. In our analyzes we added gender as a covariate, in order to compensate for these differences, however, they might still be of influence. A replication in an independent sample is recommended. Third, the design of this study was cross-sectional, so we cannot make any conclusions regarding causality. Given that we hypothesize that cognitive insight influences self-stigma over time, it would be recommended to investigate this longitudinally. Fourth, we did not have information on whether participants were, or had been, receiving treatment. This may have influenced cognitive insight levels, as cognitive insight was intact in the clinical sample and UHR sample.

Given the abundance of evidence on the negative correlates of self-stigma (6–15), clinicians should focus in treatment on self-stigma as soon as possible, to prevent individuals at UHR for psychosis form further alienation or development of more self-stigma and its negative correlates. In a review (46) on self-stigma interventions six effective interventions were found [Healthy self-concept (47, 48), Self-stigma reduction program (49), Ending self-stigma (50), Narrative enhancement and cognitive therapy (51–54), Coming out proud (55, 56), and Anti-stigma photo-voice intervention (57)], key ingredients appear to be psychoeducation, cognitive restructuring, and focus on telling one’s narrative. More recently evidence was found for the effectiveness of interventions aiming to enhance self-compassion or those based on acceptance and commitment therapy (58). Some self-help interventions are available, but the results are mixed, in line with recommendations for treatment interventions focus should be on psychoeducation and acceptance and commitment therapy (59). None of these interventions have been tested in the UHR population, which should be a focus of future research. Besides a direct focus on self-stigma, interventions aiming to improve social functioning or to prevent drop out from social roles, like Supported Employment and Supported Education, could also be useful, they seem effective in the UHR group and may contribute to lowering stigma (60).

To conclude, our results suggest that self-stigma is already present in the UHR of psychosis phase, to a similar degree as in individuals with a diagnosis of an SSD. Cognitive insight in individuals at UHR of psychosis appears to be intact, and those with more cognitive insight experience more self-stigma. Future studies should explore the nature of this association longitudinally. Overall, the findings of the current study imply that pre-emptive interventions targeting self-stigma are needed early on in manifestation of psychotic illness, preferably in the UHR phase.

## Data availability statement

The raw data supporting the conclusions of this article will be made available by the authors, without undue reservation.

## Ethics statement

The studies involving human participants were reviewed and approved by Medical ethics committee (METc) of the University Medical Center Groningen. The patients/participants provided their written informed consent to participate in this study.

## Author contributions

GP, RD, SJ, PL, and NB contributed to conception and design of the study. RD, SJ, BS, ME, TG, and DK collected the data and organized the database. BS and ME performed the statistical analysis and wrote the first draft of the manuscript. PL, IH-O, NB, and GP wrote sections of the manuscript. All authors contributed to manuscript revision, read, and approved the submitted version.

## Funding

Funding was received from Fonds NutsOhra for the original RCT in the Metacognitive Reflection and Insight Therapy (MERIT) for patients with schizophrenia study (30). Fonds NutsOhra had no influence on the study or dissemination of the results.

## Conflict of interest

The authors declare that the research was conducted in the absence of any commercial or financial relationships that could be construed as a potential conflict of interest.

## Publisher’s note

All claims expressed in this article are solely those of the authors and do not necessarily represent those of their affiliated organizations, or those of the publisher, the editors and the reviewers. Any product that may be evaluated in this article, or claim that may be made by its manufacturer, is not guaranteed or endorsed by the publisher.
